# Pleiotropic Effect of IL-6 Produced by B-Lymphocytes During Early Phases of Adaptive Immune Responses Against TB Infection

**DOI:** 10.3389/fimmu.2022.750068

**Published:** 2022-01-27

**Authors:** Irina Linge, Anastasiya Tsareva, Elena Kondratieva, Alexander Dyatlov, Juan Hidalgo, Ruslan Zvartsev, Alexander Apt

**Affiliations:** ^1^ Laboratory for Immunogenetics, Department of Immunology, Central Institute for Tuberculosis, Moscow, Russia; ^2^ Department of Cellular Biology, Physiology and Immunology, Faculty of Biosciences, Institute of Neurosciences, Universitat Autònoma de Barcelona, Barcelona, Spain; ^3^ Center for Precision Genome Editing and Genetic Technologies for Biomedicine, Engelhardt Institute of Molecular Biology, Russian Academy of Sciences, Moscow, Russia

**Keywords:** tuberculosis, B-cells, IL-6, lung inflammation, T cells

## Abstract

The role of B cells migrating to the lung and forming follicles during tuberculosis (TB) inflammation is still the subject of debate. In addition to their antibody production and antigen-presenting functions, B cells secrete different cytokines and chemokines, thus participating in complex networks of innate and adaptive immunity. Importantly, lung B-cells produce high amounts of the pleiotropic gp130 cytokine IL-6. Its role during TB infection remains controversial, partly due to the fact that IL-6 is produced by different cell types. To investigate the impact of IL-6 produced by B cells on TB susceptibility and immune responses, we established a mouse strain with specific IL-6 deficiency in B cells (CD19cre-IL-6fl/fl, B-IL-6KO) on the B6 genetic background. Selective abrogation of IL-6 in B cells resulted in shortening the lifespan of TB-infected B-IL-6KO mice compare to the wild-type controls. We provide evidence that at the initial TB stages B cells serve as a critical source of IL-6. In the lung, the effect of IL-6 deficiency in B cells is associated rather with B and T cell functioning, than with macrophage polarization. TB-infected B-IL-6KO mice displayed diminished sizes of B cells themselves, CD4^+^IFN-γ^+^, Th17^+^, and CD4^+^CXCR5^+^ follicular T cell populations. The pleiotropic effect of B-cell-derived IL-6 on T-cells demonstrated in our study bridges two major lymphocyte populations and sheds some light on B- and T-cells interactions during the stage of anti-TB response when the host switches on a plethora of acquired immune reactions.

## Introduction

Tuberculosis (TB) remains a global health problem, affecting about 10 million people and claiming about 1.2 – 1.5 million lives annually (1). Macrophages and T cells are traditionally considered as the main protective cells during anti-TB immune responses (2, 3), while the role of B-cells, whose prominent feature is the formation of B-cell follicles (BCFs) in the tuberculous lung in close vicinity to TB granuloma, is still the subject of debates (4–6). In addition to antibody production, B cells apparently play many different roles in anti-TB response by producing cytokines/chemokines, serving as antigen-presenting cells, and interacting with other immune cells during lung inflammation [reviewed (4, 6, 7)]. Regarding B-cell cytokine production, it was shown in animal models that B cells from TB-affected lung secrete almost no IL-2, high amounts of IL-6, IL-11, and IL-17, and low amounts of IL-10, IFN-γ, and TNF-α (8, 9). Although produced in low amounts, TNF-α from B cells is involved in the formation of lung BCFs in TB-infected mice (10).

Both in mice and TB patients, it was demonstrated that B cells display a STAT1-centered gene expression signature, produce type 1 interferons (11) and secrete IL-35 (12, 13). Regarding TB pathophysiology, it was emphasized that type 1 interferons polarize macrophages toward the anti-inflammatory M2 phenotype (11), whilst IL-35 production by B cells is associated with down-regulated Th1/Th17 response and an increase of the FoxP3^+^ Treg population (13). Overall, the physiology of B-cell-secreted cytokines in TB infection is only superficially characterized. In particular, specific activities of IL-6 produced by B-cells remain obscure despite its abundance in TB-infected lungs (8, 9).

IL-6 is a pleiotropic gp130 cytokine that is involved in several immune reactions and displays pro- and anti-inflammatory activity depending upon its cellular sources and the phase of the inflammatory process (14). IL-6 is produced by a variety of cells, including T- and B-cells, macrophages, and CD11c^+^ cells. Infection of mice with systemic *il6* gene knockout (KO) with a high dose of virulent *M. tuberculosis via* intravenous (i. v.) route demonstrated a markedly elevated susceptibility, decreased IFN-γ, and increased IL-4 production compared to the wild type (WT) mice (15). On the other hand, aerosol infection in a similar experimental setting showed much milder differences in the disease progression between KO and control mice. In addition, although primary IFN-γ production in IL-6KO mice was diminished, mycobacteria-specific memory T cell response remained intact (16). Summarizing, a complete abrogation of IL-6-dependent immune reactions during TB course looks rather detrimental, and in patients with pulmonary TB IL-6 levels in sera are elevated compared to healthy donors (17, 18).

Despite insufficient information available, it is reasonable to assume that the role of IL-6 produced by different cell types in TB pathogenesis is different, as it is firmly established for another key inflammatory cytokine associated with anti-TB response, TNF-α (19). Among scattered observations, it is worth mentioning that *M. tuberculosis*-infected macrophages produce IL-6, which inhibits responsiveness to IFN-γ of non-infected macrophages (20). In the context of the present study it should be mentioned that IL-6 has a prominent role in CXCR5^+^ T follicular helper (Tfh) cell differentiation (21), which, in turn, is important for “correct” co-localization of T cells and macrophages within TB foci, providing an optimal anti-mycobacterial response (22).

Here, using a mouse strain bearing B-cell-specific knockout mutation in the gene for IL-6 on B6 genetic background, we evaluated in the aerosol mouse TB model the contribution of IL-6 produced by B cells to infection severity/susceptibility in general and some parameters of cellular immunity in particular.

## Materials and Methods

### Mice

Mice of the C57BL/6JCit (B6) strain (hereafter – WT) were bred under conventional, non-SPF conditions at the Animal Facilities of the Central Institute for Tuberculosis (Moscow, Russia), in accordance with the guidelines from the Russian Ministry of Health # 755, US Office of Laboratory Animal Welfare (OLAW) Assurance #A5502-11. Water and food were provided *ad labium*. Female mice 10-12 weeks of age at the beginning of experiments were used. All experimental procedures were approved by the Institutional Animal Care and Use Committee (IACUC), protocols 2, 3, 7, 10 of March 6, 2019.

Mice with ablation of IL-6 in B cells (hereafter – B-IL-6KO) were generated by crossing IL-6^flox/flox^ mice (23) with CD19-Cre knock-in mice (CD19^cre/cre^), which, together with mice with systemic IL-6 knockout (IL-6KO) were kindly provided by Prof. S. A. Nedospasov. To generate B-IL-6KO mice, parental IL-6^flox/flox^ and CD19^cre/cre^ strains were mated, and IL-6^flox/-^CD19^cre/-^ heterozygous littermates were backcrossed to the parental IL-6^flox/flox^ strain. After PCR genotyping, females bearing the IL-6^flox/flox^CD19^cre/-^ genotype were selected for further experimentation. Mice were genotyped by the genomic PCR using tail DNA. Primers for the CD19-Cre transgene CD19.8 5′-AATGTTGTGCTGCCATGCCTC-3′, CD19.9 5’- GTCTGAAGCATTCCACCGGAA-3’; lck2 5’- AATGTTGCTGGATAGTTTTTACTGC-3’; primers for the IL-6flox transgene F: 5’- CCCACCAAGAACGATAGTCA-3’; R1: 5’- GGTATCCTCTGTGAAGTCTCC-3’; R2: 5’-AGCACTTTATTGGGCTCTATACA-3’; primers for the IL-6KO F: 5’- ACCGCTATGAAGTTCCTCTC-3’; R 5’- CCAACCAACCCTACCTAGA-3’.

### Infection

**Figure 1:**
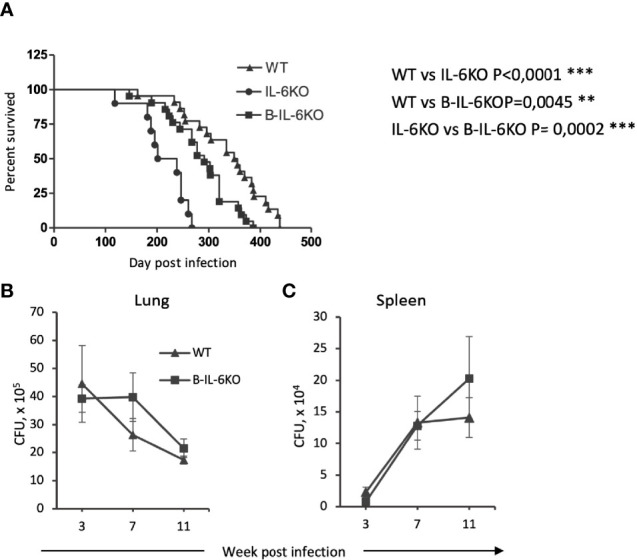
Mice lacking *il6* expression in all cells (IL-6KO), or in B-cells (B-IL-6KO), have a shorter life span after TB infection compared to control animals. **(A)** Survival time of mice of the three strains after aerosol infection with *M. tuberculosis* H37Rv, ~100 CFU per mouse. Data from two identical experiments were summarized, N = 10-25 mice for groups, log-rank test. **(B, C)** Mycobacterial counts in lungs and spleens of infected WT and B-IL-6KO mice at 3, 7 and 11 weeks post infection. Data from one of two similar experiments are displayed as the mean ± SD, 4-5 mice per group per time-point. No significant differences between the groups.

Mice were infected with ~10^2^ CFU of virulent *M. tuberculosis* strain H37Rv (sub-strain Pasteur) using the inhalation Exposure System (Glas-Col, Terre Haute, IN) exactly as previously described (24). At different time points post challenge (as indicated in **Figure 1**), mice were euthanized by the thiopental (Biochemie GmbH, Vienna, Austria) overdose.

### CFU Counts and Survival Time

At indicated time points, spleens were homogenized in 2.0 ml of sterile saline and identical lobes of the right lungs from individual mice were digested in collagenase-DNase 1 mixture as described below, except the presence of antibiotics. 10-fold serial dilutions of 0.2 ml samples were plated on Dubos agar (Difco) and incubated at 37°C for 20–22 days before CFU were counted. Survival time was monitored daily starting month 2 post-infection.

### Lung Cell Suspensions

Two upper right lung lobes from individual mice were enzymatically digested exactly as described previously (25). Two bottom right lobes were used for RNA purification. Briefly, blood vessels were washed out by perfusion with 0.02% EDTA-PBS through the right ventricle and cut vena cava, lungs removed, sliced into 1-2 mm^3^ pieces, and incubated at 37°C for 90 min in supplemented RPMI-1640 containing 200 U/ml collagenase and 50 U/ml DNase-I (Sigma, MO). Single-cell suspensions from 4 to 5 mice were obtained individually, washed twice in HBSS containing 2% FCS and antibiotics, and 3 x 10^5^ cells per sample were used for surface phenotyping. 1 x 10^6^ lung cells from each mouse were cultured overnight (16-17 hours total) in the absence (control) or in the presence of 10µg/ml mixture of mycobacterial antigens (cultural filtrate was kindly provided by Dr. Vadim Avdienko, CIT) and GolgiPlug (BD Bioscience) for the last 12 hours of incubation for further evaluation of intracellular cytokines. Cultural filtrate was obtained by culturing *M. tuberculosis* as a biofilm on the surface of the protein-free Sauton’s medium for 4 weeks, followed by precipitation of mycobacteria at 3,000g for 20 min, concentration of the protein content in the liquid phase using Amicon cell device, and bringing the final protein concentration to 1mg/ml with sterile saline.

### Flow Cytometry

Single-cell suspensions were obtained from individual mice and cell phenotypes were analyzed by flow cytometry as described previously (25) using the FACS Calibur machines (BD Biosciences). The gating strategy is displayed in **Figure S1**. The following labeled monoclonal antibodies were used: CD4-PerCp (dilution 1:300), CD4-AF488 (dilution 1:200), or CD4-PE (dilution 1:200) (all GK1.5, BioLegend), CXCR5-Fitc (L138D7, BioLegend, dilution 1:100), CD19-AF647 (6D5, BioLegend, dilution 1:400), B220-APC (RA3-6B2, BioLegend, dilution 1:400), F4/80-AF-488 (BM8, BioLegend, dilution 1:200), Ly6G-PE (1A8, BD Biosciences, dilution 1:200), CD44-FITC (IM7, BioLegend, dilution 1:150), CD62L-PE (MEL-14, BioLegend, dilution 1:200), CD11b-biotine (M1/70, dilution 1:100) with subsequent SAv-PerCP (BioLegend, dilution 1:500) staining. For intracellular staining of the lung cells, BD Fixation/Permeablization Kit (BD Biosciences) was used due to the recommendations. Briefly, after 16-17 hours of *in vitro* cultivation in the presence of mycobacterial antigens, cells were harvested, stained first for surface antigens, then fixed with Cytofix/cytoperm buffer for 30 minutes and then stained for intracellular cytokines with IFN-γ-PE (BD Biosciences dilution 1:150) and IL-17-PerCp (TC11-18H10.1, BioLegend, dilution 1:150) antibodies. Results are presented as mean ± SD.

### Lung Pathology and Immunohistochemistry

Left lungs were frozen at the regimen of –20°C to –60°C temperature gradient in the electronic Cryotome (ThermoShandon, UK), and serial 10μm-thick sections were made across the widest area. For visualization of lung pathology, sections were fixed with ice-cold acetone and stained with hematoxylin-eosin. For IHC, lung cryosections were fixed with 1% PFA, blocked with 10% donkey serum, and incubated with rabbit anti-mouse primary anti-iNOS (NovusBio) pAB, rat anti-mouse primary B220 (IgG2a, clone RA3-6B2, eBioscience), rabbit anti-mouse primary anti-ARG pAB (NovusBio), rabbit anti-mouse primary anti-CD3 (clone SP7, ThermoScientific) for 1 h at room temperature. Afterwards, preparations were incubated with secondary donkey anti-rabbit IgG-Cy3, donkey anti-rat IgG-AF488 or anti-rabbit IgG-AF488 pAB for 1 h at the room temperature. Slides were preserved using ProLong Gold anti-fade reagent with DAPI (Invitrogen-Life Technologies) before visualization using the Zeiss Axioskop40 microscope and AxioCam MRc5 AxioVisio 4 camera (Carl Zeiss, Berlin, Germany).

### RNA Purification, cDNA Synthesis and Gene Expression Evaluation

Identical lobes of the right lungs from 4-5 individual mice per group per time point were isolated and immediately put into RNA lysis buffer. Total RNA was isolated using the commercial SV Total RNA Isolation System (Promega). Reverse transcription of mRNA was performed using oligo(dT) primers, dNTP mix, M-MLV RT and RNasin^®^ (Promega). Quantitative real-time RT-PCR (qPCR) of cDNA was performed using qPCRmix-HS SYBR (Evrogen) and cfx-96 Real-Time PCR Detection System (BioRad). The following primers were used:

*hprt*: F 5′-GTGATTAGCGATGATGAACCAG-3′,
R 5′- CAAGTCTTTCAGTCCTGTCCA-3’;
*ifng*: F 5′-CATAAGCGTCATTGAATCACAC-3′,
R 5′-GGTTGTTGACCTCAAACTTGG-3’;
*il6*: F 5′-TCTATACCACTTCACAAGTCG-3′,
R 5′-TAGGCAAATTTCCTGATTATATCCA-3’;
*il11*: F 5′-ACATGAACTGTGTTTGTCGC-3′,
R 5′-ATCGGGTTAGGAGAACAGC-3′;
*il17a*: F 5′-CCAGAATGTGAAGGTCAACC-3′,
R 5′-TTCATTGCGGTGGAGAGTC-3’;
*il21*: F 5′-AAACTCAAGCCATCAAACCCT-3′,
R 5′-AGGAAGGGCATTTAGCTATGTG-3;
*inos*: F 5’-GGCAGCCTGTGAGACCTTTG -3’,
R 5’-GCATTGGAAGTGAAGCGTTTC -3’;
*arg1*: F 5’-GGAATCTGCATGGGCAACCTGTGT-3’,
R 5’-AGGGTCTACGTCTCGCAAGCCA-3’;
*fizz*: F 5’-TTGCCAATCCAGCTAACTATCCC-3’,
R 5’-CTCCCAAGATCCACAGGCAA-3’;
*ym1*: F 5’-CTGAAAGACAAGAACACTGAGC-3’,
R 5’-ATGGCACTGAACGGGGC-3’;
*stat1*: F 5’-TGAGACTGTTGGTGAAATTGC-3’,
R 5’-CTTCCGAAATCCTTTAACTGTG-3’
*stat3*: F 5′-AACTTAATGAAGAGTGCCTTCG-3′,
R 5′-TCAACTCAGGAAATTTGACCAG-3’

Relative expression levels were calculated by normalizing levels for genes of interest to that of *hprt* using the 2^–ΔΔCt^ method.

Statistical analysis was performed using GraphPadPrism7 software. Representative data from one of two identical experiments are displayed, except survival curves for which combined results from two independent experiments were summarized. The log-rank test for survival and One- or Two-way Anova with Tukey post-test for multiple comparison for other experiments were used. *P* < 0.05 was considered statistically significant.

## Results

### Both Systemic and Conditioned Deficiency in IL-6 Production Increases Severity of TB Infection

In early studies, it was shown that in mice with genetically disrupted *il6* gene in all cells (systemic IL-6KO) i. v. challenge with virulent *M. tuberculosis* resulted in a more rapid mortality compared to the wild type mice (15). Similar work utilizing the aerosol infection route reported an increase in the lung mycobacterial counts in IL-6KO mice; however, mortality was not assessed in this study (16). Thus, we evaluated the influence of systemic and B-cell-specific IL-6 deficiency on the most prominent features of chronic mycobacterial infections, the life span of mice, and mycobacterial multiplication in their organs. As shown in **Figure 1A**, both systemic and B-cell-restricted IL-6 elimination significantly decreased survival time of the infected animals compared to controls, although selective IL-6 defect in B-cells resulted in a less severe phenotype. Unexpectedly, no influence of KO mutations on lung (**Figure 1B**) and spleen (**Figure 1C**) CFU counts was observed, suggesting that a more rapid mortality in IL-6-deficient animals was not due to higher mycobacterial loads in their organs.

### IL-6 Deficiency in B Cells Alters the Early Mycobacteria-Specific Response of CD4^+^ T Cells

**Figure 2:**
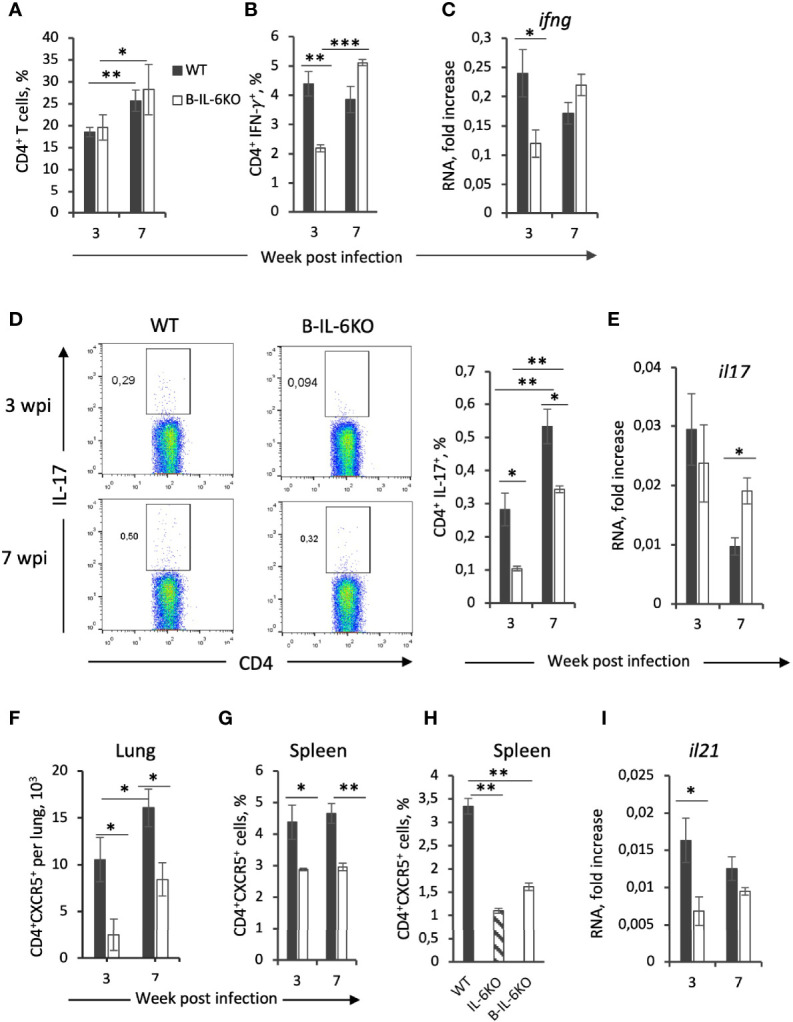
Characteristics of T-cell responses of *M. tuberculosis*-specific CD4^+^ T cells from B-IL-6KO and WT mice. **(A)** Frequency (%) of total CD4^+^ T cell population in the lung of IL-6B and WT mice, revealed by flow cytometry in 3 and 7 weeks post-infection. **(B, D)** Single cell lung suspensions were cultivated overnight in the presence or absence (control) of mycobacterial antigens and subsequent intracellular cytokines IFN-γ **(B)** and IL-17 **(D)** produced by CD4^+^ T cells were evaluated by flow cytometry. **(C, E, I)** qPCR analysis of *ifng*, *il17*, and *il21* mRNAs in lungs at week 3 and 7 post infection. Expression of each gene was normalized to *hprt*. **(F–H)** The amount of CD4^+^CXCR5^+^Tfh cells in lungs **(F)** and frequency within CD4^+^ population in spleens of infected **(G)** and non-infected **(H)** B-IL-6KO and WT animals were assessed by flow cytometry. Results are expressed as mean ± SD, 4-5 mice per group per time-point. The results from one of two similar experiments are displayed. *P < 0.05; **P < 0.01; ****P < 0.0001.

Production of IFN-γ by CD4^+^ T cells is considered critical for protective immunity against *M. tuberculosis* (2). Since there is evidence that in IL-6KO mice general production of IFN-γ is decreased (15, 16), we evaluated how the B-cell-produced IL-6 influences IFN-γ production in the lungs. After culturing overnight *in vitro* in the presence of mycobacterial antigens, IFN-γ response in CD4^+^ T lung cells from infected WT and B-IL-6KO mice was assessed by flow cytometry (intracellular staining). Additionally, we evaluated the overall expression level of the *ifng* gene in the lung tissue by qPCR. The proportion of CD4^+^ T cells infiltrating lungs increased significantly during the course of infection in mice of both strains, and their percentage did not differ between strains at weeks 3 and 7 post-infection (**Figure 2A**). However, in B-IL-6KO mice, at week 3 post-infection the proportion of IFN-γ-positive lung CD4^+^ cells was significantly lower than in control animals (**Figure 2B**). In addition, at this time point the expression level of *ifng* gene in the lung was also decreased in B-IL-6KO mice (**Figure 2C**). These differences disappeared by the week 7 post challenge (**Figure 2**).

IL-6 is important for the development and maintenance of a balanced Th17 immune response (26), whose role in TB is ambiguous, although several studies suggested the importance of Th17 population for the effective immune response against TB (27, 28). Given that in mice bearing either genetically disrupted *il6*, or lacking the gp130 receptor in T cells, development of IL-17-producing Th17 cells is decreased (29), we measured whether IL-6 deficiency in B-cells could affect the mycobacteria-specific Th17 population. Lung cells from mice infected for 3 and 7 weeks were cultured overnight in the presence of mycobacterial antigens and IL-17 production by CD4^+^ T cells was assessed by flow cytometry. Even though the overall frequency of mycobacteria-specific CD4^+^IL-17^+^ cells was lower than 1%, the CD4^+^IL-17^+^ population was significantly smaller in B-IL-6KO mice at both time points (**Figure 2D**). However, the expression level of the *il17* gene in the lung was equal in the two mouse strains at week 3 or even slightly increased in B-IL-6KO mice at week 7 post infection (**Figure 2E**).

IL-6 is crucial for differentiation of follicular CXCR5^+^ Tfh, which are required for the “correct” co-localization of T cells and macrophages in the manner optimal for TB control (22). Given that during TB course B cells migrate to the lung and form BCFs that possess all prominent features of BCFs of secondary lymphoid organs, including the presence of CD4^+^CXCR5^+^ Tfh cells (22, 30–32), we assumed that the absence of IL-6 in B cells may decrease the content of these regulatory T-cells during infection. Indeed, both in lungs and spleens of B-IL-6KO infected mice the proportion of CXCR5^+^ CD4^+^ Tfh-cells was significantly lower than in their WT counterparts (**Figures 2F, G**). Moreover, even before infection, both total and B-cell-specific IL-6 deficiency resulted in a significantly diminished size of this important T-cell population in spleen (**Figure 2H**, such an experiment is technically infeasible with non-infected lung tissue). In addition, at the early stage of infection lung cells of B-IL-6KO mice expressed significantly less *il21* mRNA (**Figure 2I**). This gene encodes IL-21, the cytokine intensively produced by Tfh cells (33, 34).

### B Cells Are an Important Source of IL-6 in the Lungs

**Figure 3:**
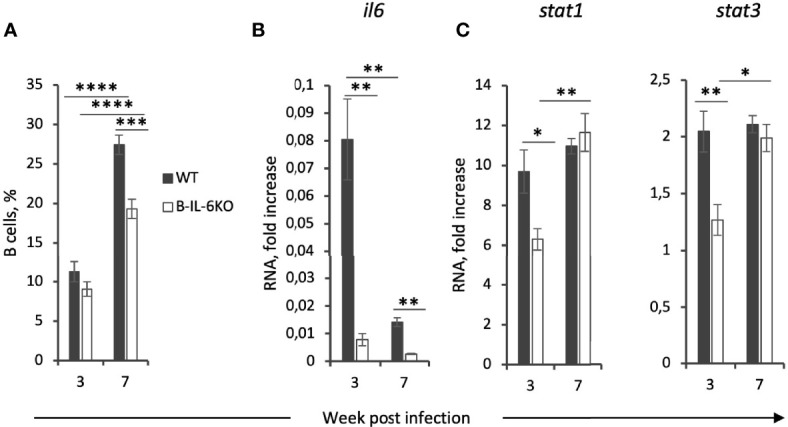
Lung B-cells are a major source of IL-6 which regulates their accumulation and the expression of IL-6-dependent *stat3* and IFN-γ-dependent *stat1* genes in the lung tissue at the early phase of infection. **(A)** The total percentile of CD19^+^ B-cells in the lungs of B-IL-6KO and WT mice at weeks 3 and 7 post infection, revealed by flow cytometry. **(B, C)** qPCR evaluation of the levels of *il6*, *stat1* and *stat3* mRNAs in the lung tissue at weeks 3 and 7 post infection. Gene expression was normalized to that of *hprt*. Results of one of two similar experiments are displayed as mean ± SD, 4-5 mice per group per time-point. *P < 0.05; **P < 0.01; ****P < 0.0001.

Since IL-6 may originate from many different cell sources, we next assessed what is the B cell input into the total IL-6 production in the lungs of infected mice. At week 3 post challenge, B cells comprise not more than 10 percent of all lung cells (**Figure 3A**). However, in B-IL-6KO mice the total amount of *il6* mRNA in the whole lung tissue appeared to be 10-fold lower compared to WT mice, suggesting that B-cells are one of the major sources of IL-6 in tuberculous lungs early after challenge (**Figure 3B**). This ratio in the *il6* gene expression was retained at least up to week 7 post infection, although the overall expression level significantly decreased by this time point (**Figure 3B**), despite substantial growth of the lung B-cell content, which also partially depended upon B-cell-provided IL-6 (**Figure 3A**). We limited our IL-6 measurements by the total mRNA assessment in freshly isolated lung tissue since many different types of lung cells produce this cytokine and require different culture conditions for optimal responses. Thus, lung T-cells from mice infected with *M. tuberculosis* or *Pseudomonas aeruginosa* readily secrete IL-6, TNF-α and IFN-γ after stimulation *in vitro* with specific antigens in the presence of splenic APC, but the response is markedly inhibited by lung macrophage-produced prostaglandin E2 (our unpublished observations). However, we expect to resolve these limitations by rigorous cell sorting and measure IL-6 production by different cells at the protein level in our further studies.

An important aspect of IFN-γ- and IL-6-mediated signaling is activation of two transcription factors, Stat-1 and Stat3, respectively (35, 36). We have measured the expression levels of corresponding genes and found that at week 3 of infection a decreased level of the *ifng* and *il6* expression is indeed accompanied by the lower expression of *stat1* and *stat3* in the lungs of B-IL-6KO compare to the WT animals (**Figure 3C**).

### B-Cell-Specific IL-6 Deficiency and Lung Pathology

**Figure 4:**
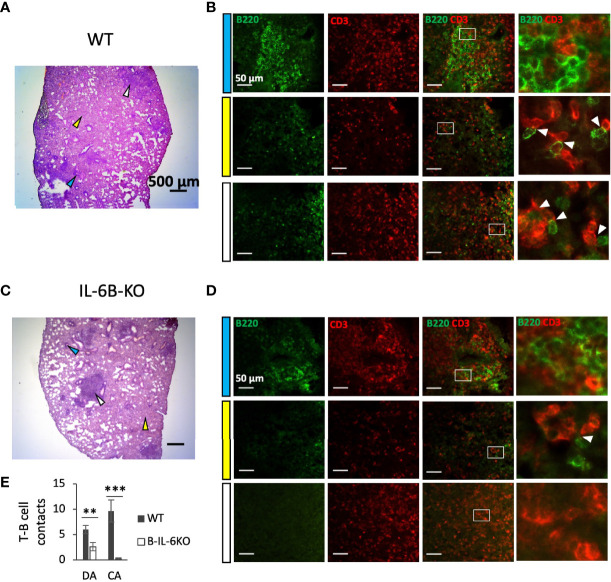
B-cell-specific IL-6 deficiency affects early lung pathology and co-localization of T- and B-lymphocytes in areas of diffuse inflammation. **(A, C)** – Representative pictures of lung pathology of WT **(A)** and B-IL-6KO **(C)** mice, 3 weeks post challenge. Arrows: blue – lymphocyte cuff near the bronchus; white – condensed inflammatory focus; yellow – diffuse infiltration. **(B, D)** – B- and T-cell co-localization within the areas indicated by arrows at **(A, C)** panels. Blue, white and yellow left-side bars correspond to the colors of the arrows at **(A, C)** panels. The right panels in **(B, D)** represent digital magnification of the squared areas, and white arrows point at co-localized B (green-stained) and T (red-stained) lymphocytes. **(E)** – Quantification of T-B cell contacts in diffuse (DA) and condensed areas (CA) of lung inflammation. Mean ± SD, counted per 10 fields. IHC staining of cryosections with a-B220 (for B cells) and a-CD3 (for T cells) antibodies. ** P < 0,01, ***P < 0,001.

To find out whether the conditioned IL-6 knockout influences TB pathogenesis, we assessed lung histopathology in B-IL-6KO and WT mice at weeks 3 and 7 post infection. There was little difference between B-IL-6KO and WT mice regarding the dynamics of TB inflammation. As shown in **Figure S2**, at week 3 post infection TB foci displayed no regular structure, whilst at week 7 we observed the development of circled granulomata with macrophage-occupied centers surrounded by lymphocyte cuffs in mice of both strains. However, synoptic pictures reflecting ratios between the areas occupied by diffuse pneumonia, condensed TB foci, and unaffected breathing tissue differed between mice of the two strains. As shown in **Figures 4A, C** and **Figure S2**, in the WT mice zones of diffuse inflammation occupied a larger proportion of the lung tissue compare to the B-IL-6KO mice at week 3 post challenge. As this was an unexpected observation, we decided to evaluate how lung-infiltrating B- and T -lymphocytes interact within the inflamed tissue. To this end, we performed the IHC staining of B- and T-cells residing within the zones of condensed and diffuse inflammation in infected lungs of the WT and B-IL-6KO mice. As shown in **Figures 4B, D**, within the lymphocyte cuffs surrounding blood vessels and bronchi B- and T-lymphocytes were located in tight vicinity in mice of both strains, making possible their physical and functional interactions, e.g., antigen presentation by B-cells. However, within the zones of diffuse pneumonia such co-localization (quite often – geminates) was substantially more frequent in the WT mice (**Figure 4E**, left). Furthermore, B-cells were practically lacking within the condensed inflammatory foci in lung parenchyma of B-IL-6KO animals (**Figure 4E**, right). A substantial increase of the area of T- and B-cell contacts in the WT mice possibly provide additional opportunities for the development of acquired immunity in the lung.

Since we obtained a histological evidence that the B-cell-produced IL-6 is involved in early lung cells interaction after TB challenge, we decided to check a possible influence of selective IL-6 deficiency on the expression of the gene for another member of the gp130 cytokine family, IL-11, highly expressed in TB susceptible mice (37). This was prompted by our earlier *in vivo* observation that IL-11 regulates both self and IL-6 production in infected lungs (38). However, no such dual effect was observed for B-cell-specific IL-6: the level of *il11* gene expression was similar in mice of the two strains (**Figure S3**).

### Macrophage Polarization

**Figure 5:**
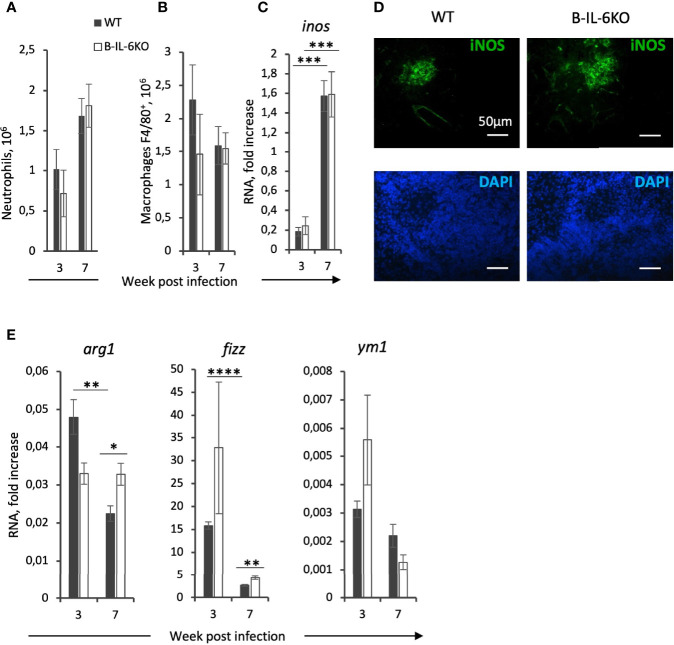
Macrophages from infected lungs display general M1-centered polarization only marginally modified by the absence of B-cell-derived IL-6. The content of Ly6G^+^ neutrophils **(A)** and F4/80^+^ macrophages **(B)** in the lungs of WT and B-IL-6KO mice, revealed by flow cytometry. **(C)** Elevation in the *inos* gene expression between weeks 3 and 7 post challenge, and **(D)** immune histochemical staining of iNOS-positive cells within TB granulomata at week 7 post infection. **(E)** qPCR measurements of *arg1, fizz*, and *ym1* RNAs in the lung tissue at weeks 3 and 7 post infection. See the legend for **Figure 3**. *P < 0,05, **P < 0,01, ***P < 0,001, ****P < 0,0001.

Infected B-IL-6KO and WT mice did not differ by the sizes of neutrophil (**Figure 5A**) and macrophage (**Figure 5B**) populations residing in their lungs. Since there is a growing body of evidence indicating that IL-6 participates in macrophage polarization toward the anti-inflammatory M2 phenotype (39–42), which is less effective for TB protection (43, 44), we tested a few parameters distinguishing M1 and M2 macrophages. Between weeks 3 and 7 of infection, in the lungs of B-IL-6KO and WT mice we observed a marked (~8-fold) and equal increase in expression of the *inos* gene (**Figure 5С**) encoding inducible NO synthase (iNOS), the enzyme playing a pivotal role in the mycobacterial killing in mice (45) and a key marker of the M1 macrophage subset. This was supported by IHC staining, visualizing high amounts of iNOS in the central zones of TB lung granulomata in mice of both strains (**Figure 5D**), whilst at week 3 post challenge iNOS-positive cells were almost undetectable in the lung tissue and absent in intact lungs (**Figure S4**). Surprisingly, whilst in B-IL-6KO lungs the level of *arg1* gene expression remained stable, it was significantly down-regulated in the WT mice between weeks 3 and 7 (**Figure 5C**), despite a higher *il6* gene expression level in their lungs at both time points. A more prominent production of arginase instead of inducible NO synthase is often regarded as a property of M2 macrophages. We observed a stable level of expression of the corresponding *arg* gene in B-IL-6KO mice, whilst in WT controls its expression sharply dropped and became significantly lower at week 7 post challenge (**Figure 5E**). A suggestively higher expression of the *fizz* gene involved in the M2 polarization pathway was also observed in B-IL-6KO mice at week 7 post infection, while the expression of *ym1* was equal in the two strains (**Figure 5E**). We also tried to assess arginase expression in lungs of mice of the two strains by IHC staining. As shown in **Figure S5**, both in WT and IL-6KO mice arginase-positive cells were present in the inflamed lung tissue in similar quantities. Thus, there was only weak evidence of the involvement of B-cell-derived IL-6 in M2 polarization, however, irrespective to the presence or absence of IL-6 produced by B-cells, M1 pathway was much more prominent during the early stage of TB infection.

## Discussion

IL-6 participates in host defense immune reactions against viral, parasitic, fungal and bacterial infections and primary immunodeficiency diseases involving IL-6 or its signaling pathways confirm these observations. Application of IL-6 inhibitors in a clinical setting demonstrated an increased risk of infections, including tuberculosis (46). However, the role of IL-6 during TB in humans remains controversial. Few studies revealed increased IL-6 concentrations in sera of patients with far-advanced pulmonary TB compared to healthy subjects (17) and elevated level of *il6* expression in peripheral blood cells from TB patients (18). Other research teams reported on either a decrease in IL-6 levels in supernatants of the whole blood cultures from patients with active compare to latent TB (47) or the lack of significant differences between these cohorts (48, 49).

IL-6 production by many different cells may potentially have different effects on inflammation, which requires the application of mouse strains with cell type-specific KO mutations for *il6* or its receptor genes for experimental dissection of the effects of IL-6 from diverse cell sources on different cells. Thus, TB infection in mice with conditional gp130 KO in macrophages and neutrophils was characterized by elevated levels of pro-inflammatory cytokines and enhanced Th1 and Th17 immune responses, along with an increased iNOS expression (50). However, these serious shifts in immune responses have not enhanced the control of mycobacterial growth, probably since gp130 is a common receptor for other cytokines, e. g., IL-11 and IL-27, and too many bystander regulatory pathways were altered in this experimental setting. An analogous approach was applied recently in the study of Ritter and coauthors (29), who used CD4^cre^gp130^loxP/loxP^ mice for studying the role of IL-6 in Th17 induction during TB when signaling from IL-6 was abrogated in CD4^+^ T cells. Their data showed that gp130-provided signaling in these cells was not absolutely essential for Th17 induction. In the present study, we demonstrate that selective elimination of IL-6 from B-cells increases susceptibility to TB in terms of the post-infection life span (**Figure 1**). However, no increase in mycobacterial CFU content in organs compare to control animals was found, in parallel with similar earlier investigations (22, 29). The fact that susceptibility to TB, as measured by mortality curves or CFU counts, not always correlate was reported previously (51). By now, there are no unambiguous explanations for the lack of correlation between CFU and mortality data.

As mentioned above, B cells migrate into TB-infected lung tissue and form BCFs in close vicinity of granulomata. CD4^+^ T cells also migrate into the lung and are readily observed within BCFs where they directly contact B-cells and proliferate (9, 52). Thus, we assessed whether the lack of IL-6 in B cells influences the establishment of mycobacteria-specific T cell responses. It appeared that IL-6 deficiency in B cells leads to a general down-regulation of the *ifng* gene expression in the lung and a decreased frequency of mycobacteria-specific CD4^+^IFN-γ^+^ T cells at the early stage of infection (**Figure 2B**). Interestingly, the frequency of CD4^+^IL-17^+^ cells was also decreased in B-IL-6KO mice (**Figure 2D**). These results are in agreement with observations made by Ritter et al. (28), who showed that the numbers of mycobacteria-specific Th17 cells in CD4^cre^gp130^loxP/loxP^ mice were decreased. This suggests that both IL-6 from B cells and IL-6/gp130 signaling in CD4^+^ T-cells are important for Th17 differentiation.

BCFs in the tuberculous lung possess all prominent features of BCFs of secondary lymphoid organs, including the presence of CXCR5^+^ Tfh cells (22, 53). Since Tfh differentiation requires both IL-6 and IL-21 (21, 54), and the number of “TB BCFs” is reduced in mice with systemic IL-6 knockout (22), we evaluated whether IL-6 exactly from B cells has an impact on Tfh content. As shown in **Figure 2**, not only the content of CXCR5^+^ Tfh cells was decreased in infected B-IL-6KO compared to control mice, but in spleens of non-infected IL-6KO and B-IL-6KO animals, the numbers of CXCR5^+^ Tfh cells also were lower than in controls. This suggests that IL-6 from B cells is required for general differentiation and maintenance of CXCR5^+^ Tfh cells. In addition, at week 3 post infection the expression level of the *il21* gene was lower in B-IL-6KO mice (**Figure 2**). Lymphocyte sources of IL-6, which triggers IL-21 production by CD4^+^ T cells (55), include B-cells, NK cells, and CD8^+^ T cells (56). Our results show that IL-6 from B cells is an important factor for local Tfh formation and IL-21 production during the early phase of infection.

It is worth mentioning that along with the infection progression (weeks 7 and 11) we observed a gradual decrease in the influence of IL-6 from B-cells onto T cell response against mycobacteria. Given that at week 3 post infection, despite an equal content of B-cells, the overall level of *il6* expression in the lung is ~10 fold lower in B-IL-6KO mice compared to controls, we may conclude that B-cells serve as a major source of IL-6 in the lung exactly at the early phase of infection. This correlates with the results obtained in nonhuman primates, showing that the depletion of B-cells prior to infection results in a decrease in general lung IL-6 production (8). Our observation that IL-6 of the B-cell origin is requisite for B- and T-cell co-localization in the lung zones distant from blood vessels and bronchi (**Figure 4**) also suggests that at the early phase of infection IL-6 from B-cells plays an important role in the development of acquired anti-TB immunity in the lung. However, it is fairly possible that when immune response is already activated and the overall IL-6 level decreases, other sources of this cytokine are sufficient for supporting the ongoing antigen-specific T cell response and may replace B-cell-produced IL-6. For example, it was reported in the context of TB at the murine type 2 diabetes background that NK-CD11c^+^ cells were the main source of IL-6 at month 6 post challenge (57). Lung B-cells mostly reside in follicles and interact with limited numbers of co-localized T-cells, whilst a larger part of the T-cell population is diffusely distributed in the lung tissue, where IL-6 from macrophages and CD11c^+^ antigen-presenting cells may serve as an alternative source. In addition, there is evidence that IL-6 production by T-cells themselves is supported by a positive autocrine regulatory loop (58).

Taken together, our results clearly indicate that the most prominent role of B-cell-derived IL-6 in anti-TB response concerns the early phase of infection – more precisely, the third week of its development – when the host starts switching on a plethora of acquired immune reactions (2). More experimentation is needed to clarify its possible role during the late phases of infection, on the background of severe lung pathology and dysfunction. Nevertheless, pleiotropic effects of B-cell-derived IL-6 demonstrated in our study provide some explanations of how B- and T-cells interact during the early phase of anti-TB responses.

## Data Availability Statement

The raw data supporting the conclusions of this article will be made available by the authors, without undue reservation.

## Ethics Statement

The animal study was reviewed and approved by Institutional Animal Care and Use Committee (IACUC), protocols 2, 3, 7, 10 of March 6, 2019.

## Author Contributions

IL, AT, AD, and EK performed experiments. RZ assisted for CD19cre-IL-6flox/flox mouse strain establishment and creation of primers for qPCR. JH established IL-6flox mouse strain. IL and AA designed experiments, discussed results and wrote the manuscript. All authors contributed to the article and approved the submitted version.

## Funding

This work was supported by the RFBR grant 19-04-00058 (to AA). Maintenance of IL-6fl/fl and CD19-Cre mice was supported by the grant 075-15-2019-1660 from the Ministry of Science and Higher Education of the Russian Federation. JH acknowledges Grant RTI2018-101105-B-100 from Ministerio de Economía y Competitividad and European Regional Development Fund. The revision of the manuscript was supported by Russian Science Foundation 22-25-00308 (to IL).

## Conflict of Interest

The authors declare that the research was conducted in the absence of any commercial or financial relationships that could be construed as a potential conflict of interest.

## Publisher’s Note

All claims expressed in this article are solely those of the authors and do not necessarily represent those of their affiliated organizations, or those of the publisher, the editors and the reviewers. Any product that may be evaluated in this article, or claim that may be made by its manufacturer, is not guaranteed or endorsed by the publisher.
