# Supplementary figures and images for: Pleiotropic Effect of IL-6 Produced by B-Lymphocytes During Early Phases of Adaptive Immune Responses Against TB Infection
Source: Front Immunol. 2022 Jan 27;13:750068. doi: 10.3389/fimmu.2022.750068 (PMC8828505; doi:10.3389/fimmu.2022.750068)

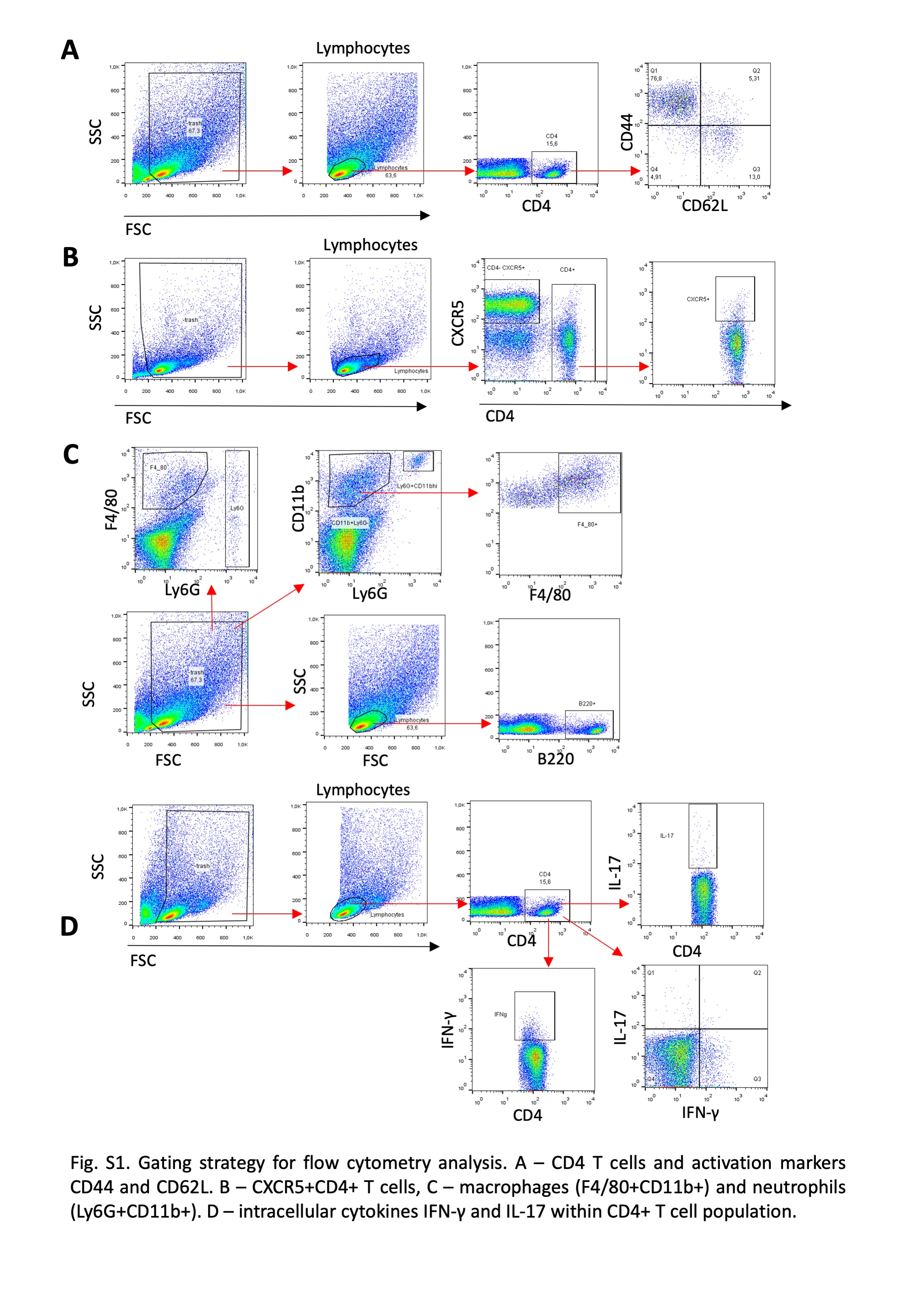

Supplement: Supplementary file 1 [file Image_1.jpeg]

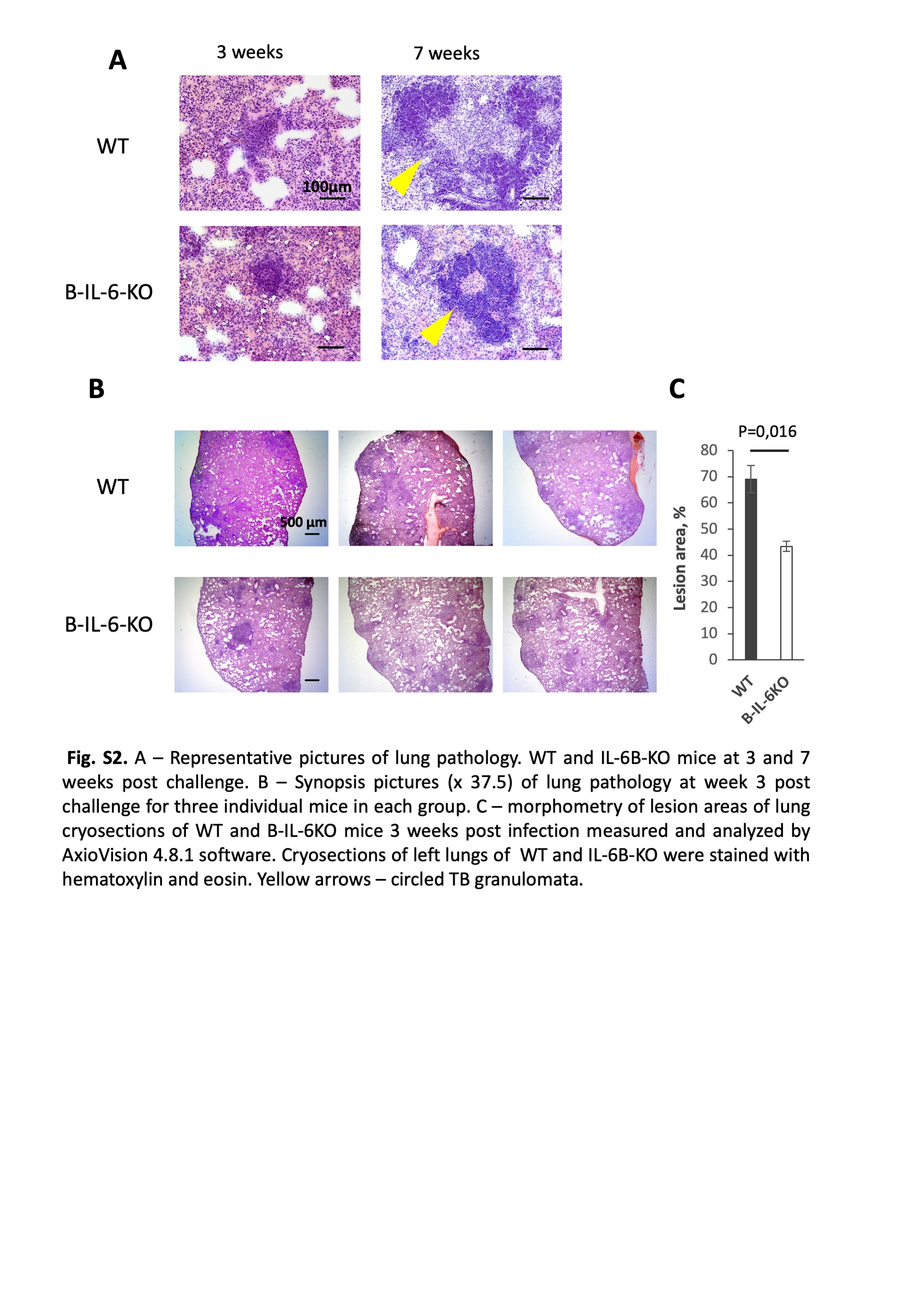

Supplement: Supplementary file 2 [file Image_2.jpeg]

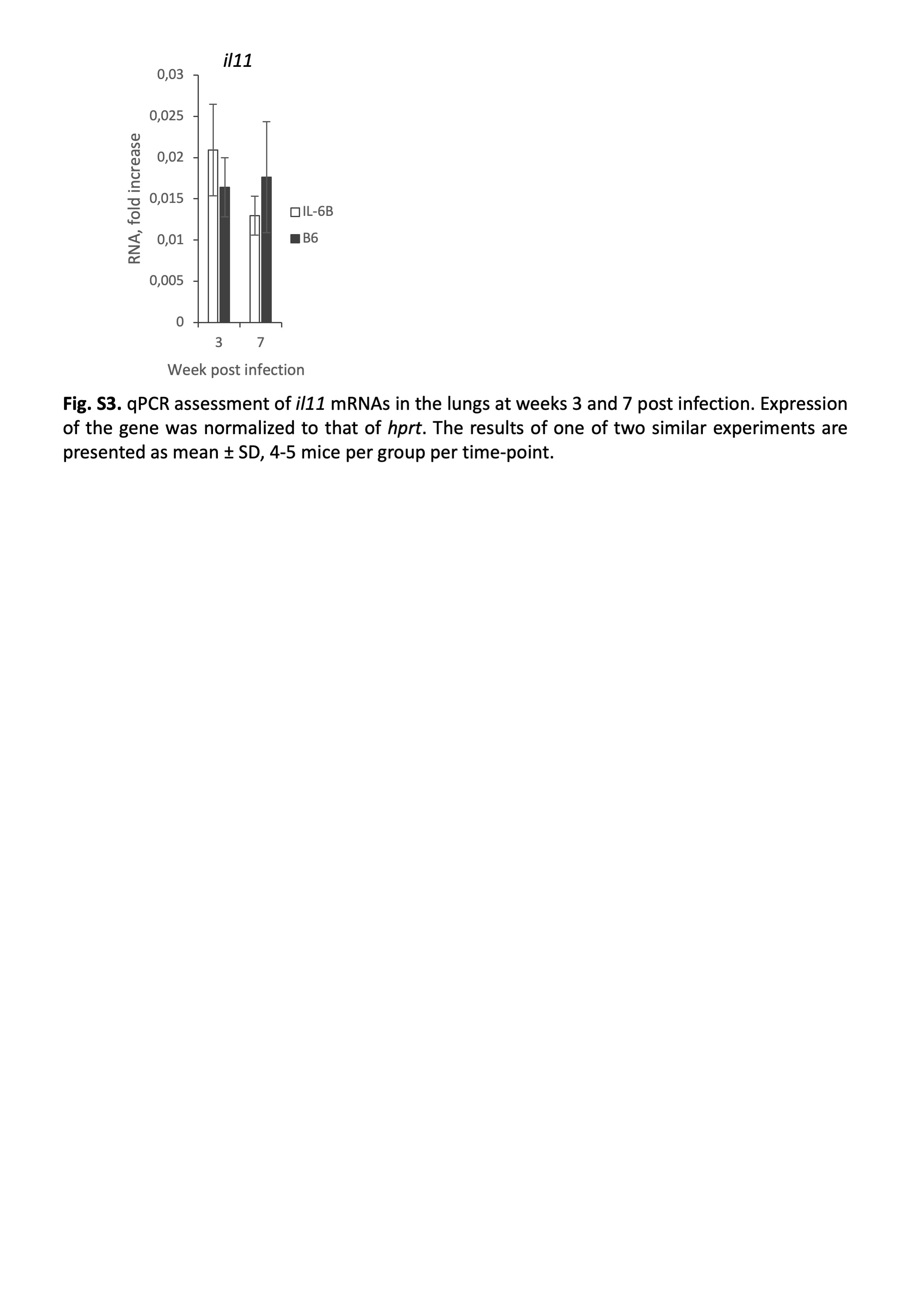

Supplement: Supplementary file 3 [file Image_3.jpeg]

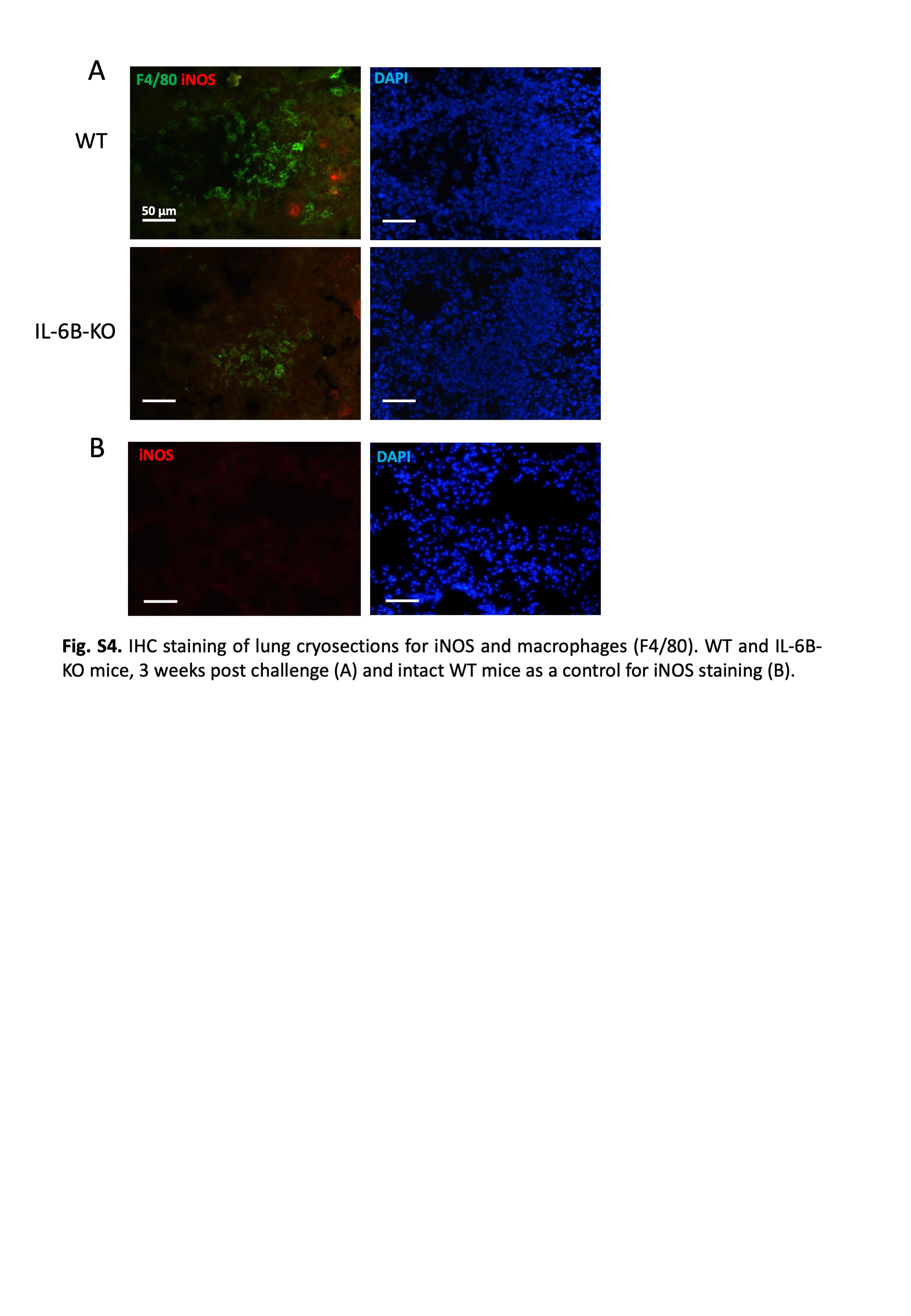

Supplement: Supplementary file 4 [file Image_4.jpeg]

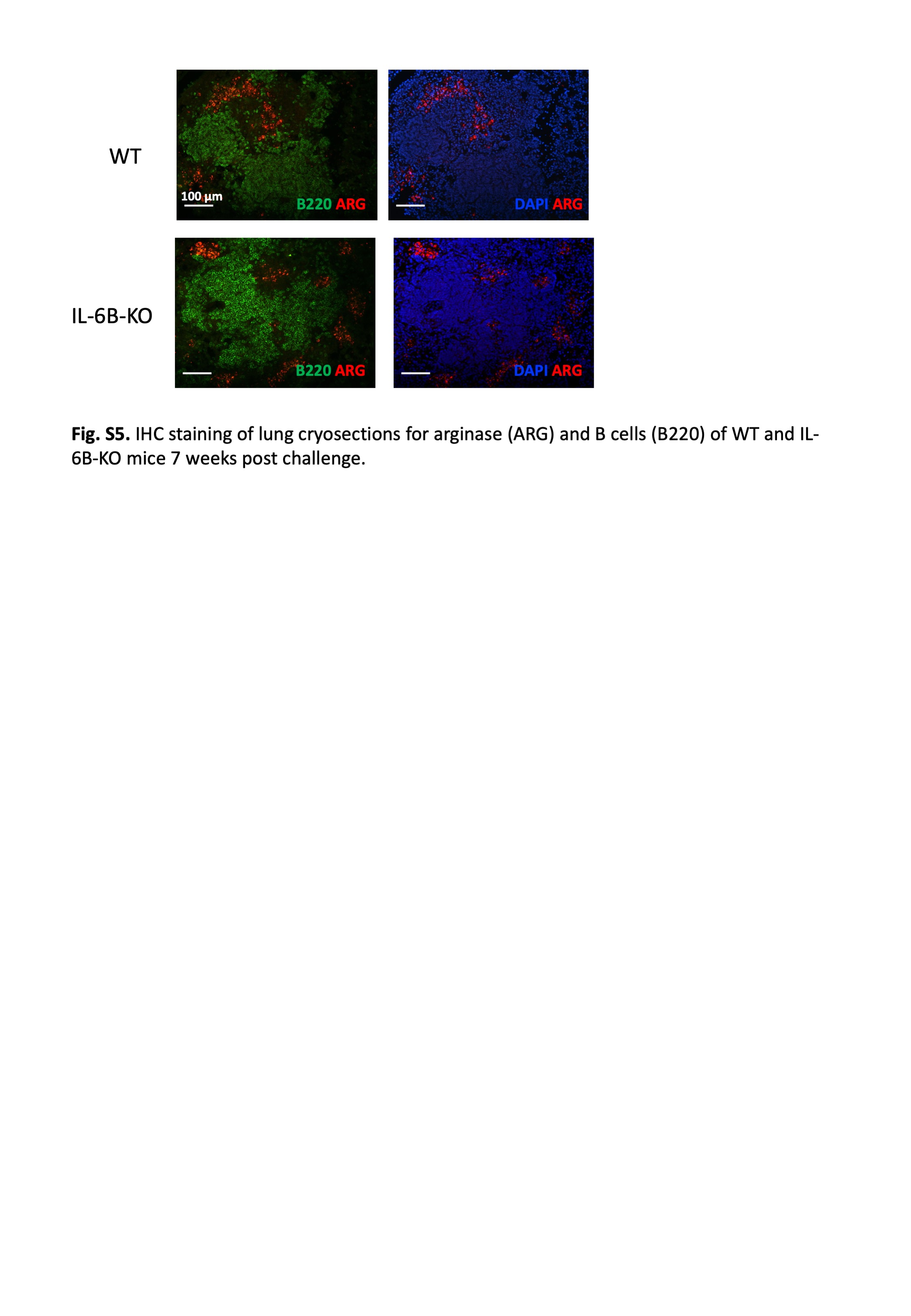

Supplement: Supplementary file 5 [file Image_5.jpeg]
